# Sequential MRI as a Diagnostic Tool for Follow‐Up of Hyaluronic Acid Dermal Filler, in a Woman Who Underwent Radiation Therapy for Oral Cancer

**DOI:** 10.1002/ccr3.70402

**Published:** 2025-04-08

**Authors:** Gloria Bettini, Ferdinando De Negri, Roberto Amore, Sergio Rexhep Tari, Antonio Scarano

**Affiliations:** ^1^ Radiology Unit Azienda USL Toscana Nord Ovest Pisa Italy; ^2^ Center of Clinical Pharmacology for Drug Experimentation Azienda Ospedaliera Universitaria Pisana Pisa Italy; ^3^ Master Course in Aesthetic Medicine, Department of Innovative Technologies in Medicine and Dentistry University of Chieti‐Pescara Chieti Italy

**Keywords:** dermal filler, facial filler, facial rejuvenation, hyaluronic acid filler (HA filler), magnetic resonance imaging (MRI)

## Abstract

The present investigation aimed to inform the radiologists about the imaging features of injectable fillers in order not to confound these with true pathology or vice versa in order not to miss true pathology obscured by filler injections. The signal of hyaluronic acid closely follows the water signal because of its composition and its hydrophilic nature and appears strongly hyperintense.

## 1 Introduction

Many types of dermal fillers, divided into resorbable and non‐resorbable, are used in aesthetic medicine. The most commonly used are calcium hydroxylapatite, hyaluronic acid, polyalkylimide, polylactic acid, and polymethylmethacrylate (PMMA) microspheres. These dermal fillers are very safe and have relatively minimal risks and side effects. Hyaluronic acid (HA) is a polysaccharide and natural compound of the extracellular matrix of all connective tissue, which gives it a high level of hydration [1]. It can affect cell metabolism and modify the organization of the actin cytoskeleton, influencing fibroblast orientation and shape [2]. Is widely used in biostimulation and facial soft tissue augmentation procedures due to its biocompatibility [3]. HA injection, whether animal‐ or bacterial‐derived, is extremely well tolerated. Its volatizing effect is lost as resorption by hyaluronidases occurs within 6–12 months, depending on the amount and type of HA‐filling agent implanted. The aesthetic effect of HA injection is generally assessed individually and visually by the physician and lasts 6 to 12 months. However, histological investigations have shown to better understand tissue modifications and demonstrate a long life span [4]. In recent years, research on dermal filler components has improved and optimized filler composition, making available long‐lasting fillers to doctors and patients. For this reason, it is becoming increasingly common for diagnostic imaging (also carried out for other reasons) to reveal the presence of fillers and the effects of the filler injections. The aim of the present case report is to evaluate the durability of the filler through MRI investigations performed to assess the health status of oral cancer patients.

## 2 Case History and Examination

A 59‐year‐old woman presented to our attention because of her routine oncologic follow‐up after a treated oral cancer. Informed consent was obtained to perform an MRI examination. The MRI imaging protocol was obtained with a 1.5 T Siemens device and included the following sequences:

1. Axial T1‐weighted, with 3.5 mm slice thickness;
2. Axial T2‐weighted, with 3.5 mm slice thickness;
3. Axial T2 TIRM, with 3.5 mm slice thickness;
4. Coronal T2 TIRM, with 3.5 mm slice thickness;
5. Sagittal T2‐weighted, with 2.0 mm slice thickness;
6. Axial DWI, with 3.5 mm slice thickness;
7. T1 VIBE FAT SAT three‐dimensional without and with contrast, with 1.0 mm slice thickness.

## 3 Methods (Differential Diagnosis, Investigation, and Treatment)

**FIGURE 1:**
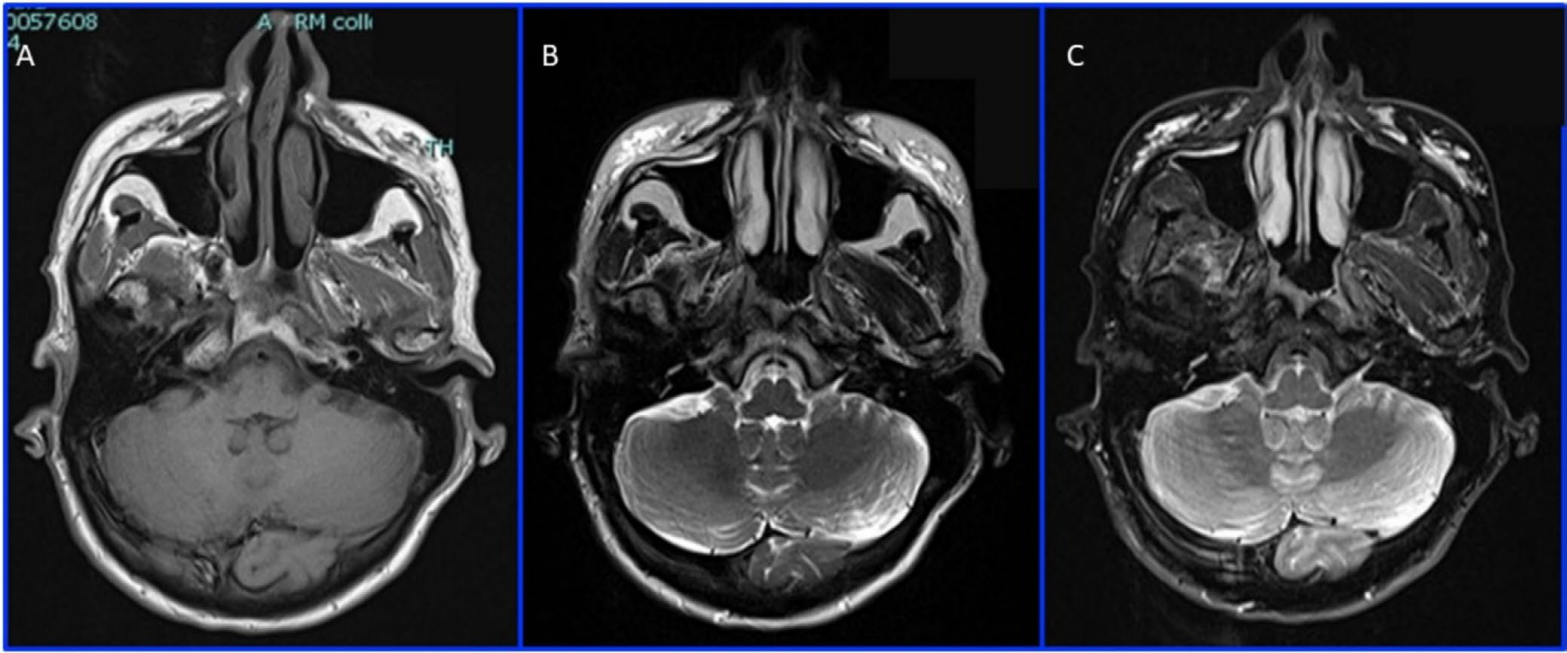
MRI: Axial T1 (A), T2 (B), and T2 TIRM (C) sequence of the III MRI, showing the presence of HA filler in the zygomatic region bilaterally; in (A) we can see the filler as low‐intensity signal areas; in (B) we identify the filler as high‐intensity signal areas; in (C) the filler is represented by very high‐intensity signal areas; we can see how T2 TIRM is the best sequence for highlighting the HA filler.

**FIGURE 2:**
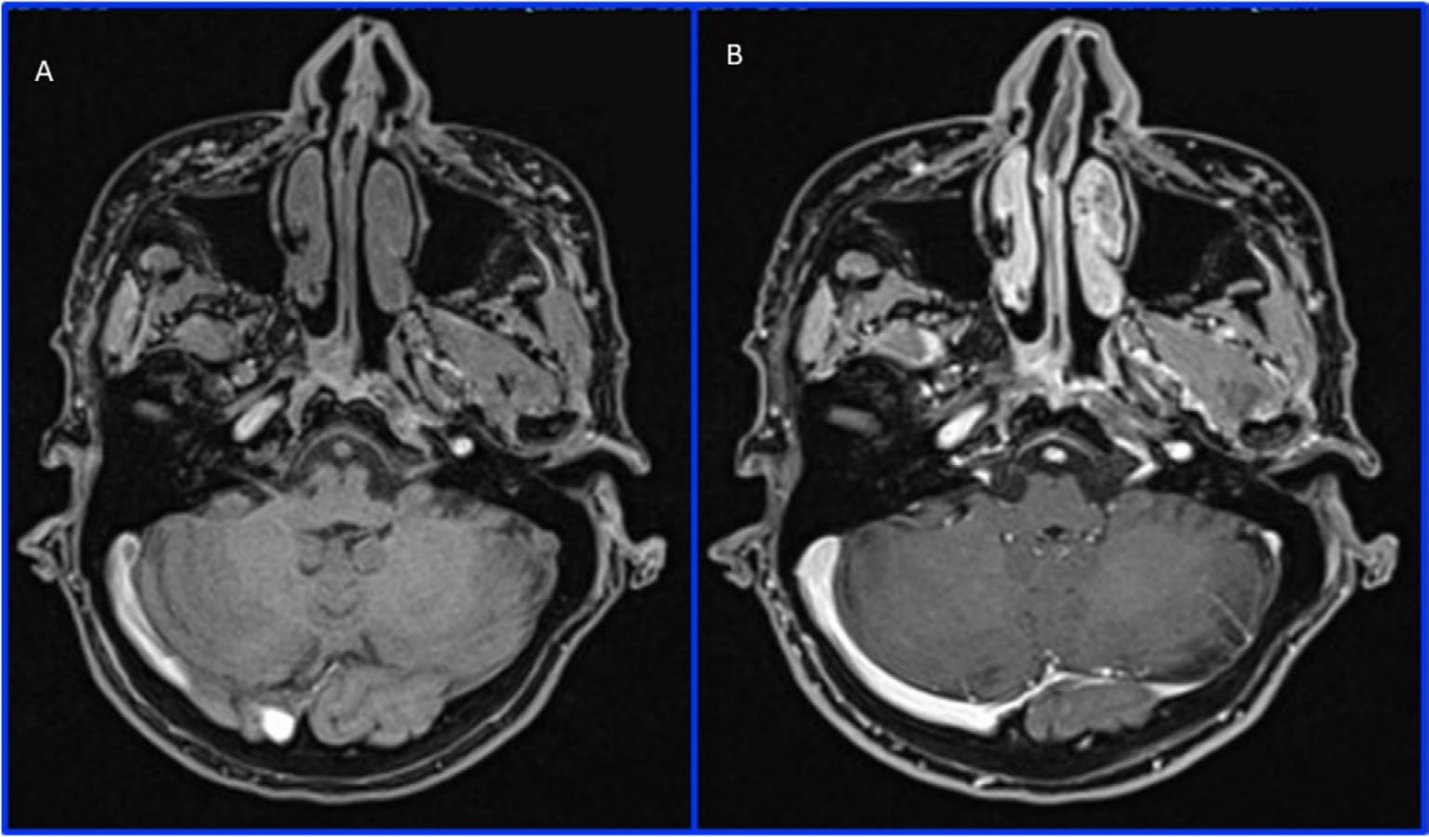
MRI: Axial T1 VIBE FAT SAT (A) e T1 VIBE FAT SAT after contrast medium administration (B) of the III MRI, showing no difference between with or without contrast images in highlighting the HA filler, which appears as low signal areas. We could not see a significant difference between the first and the second MRI (performed more than 6 months later), and in the third MRI, the filler was also well represented.

**FIGURE 3:**
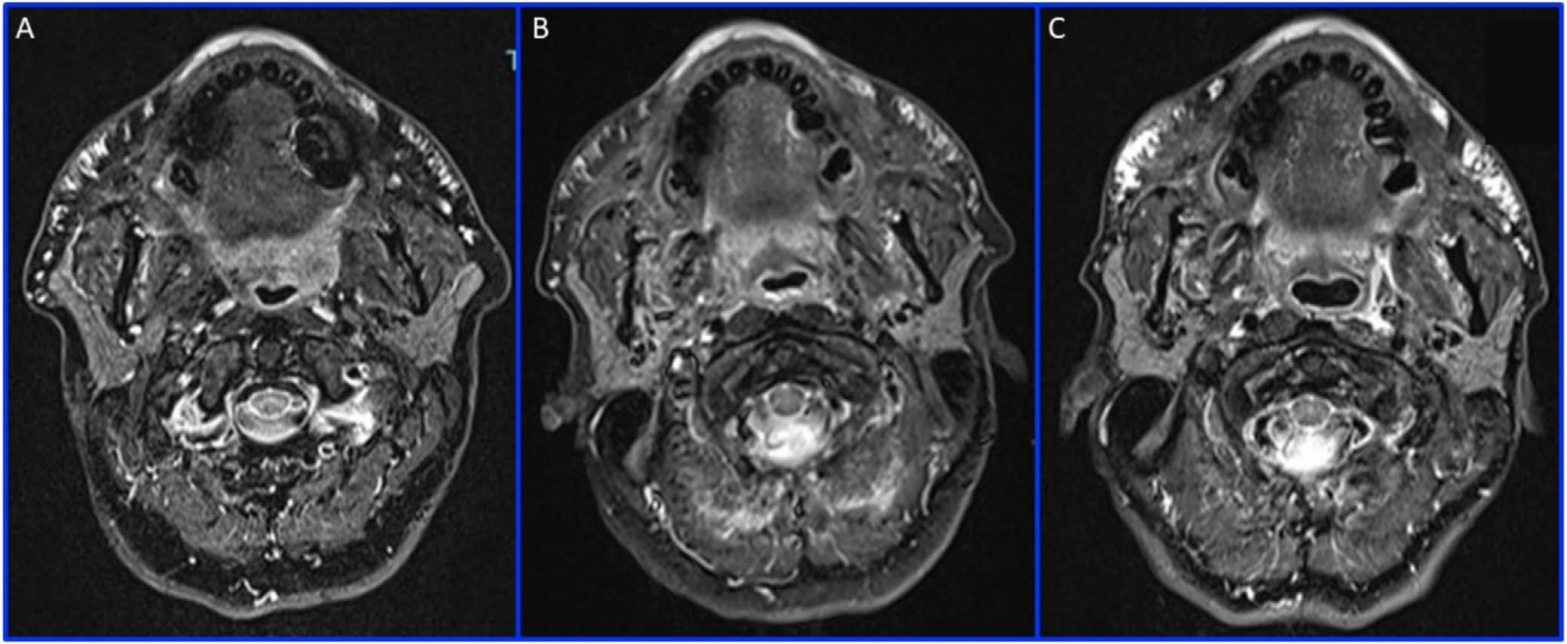
MRI: Axial T2 TIRM in I (A), II (B), and III (C) MRI, showing the persistence of HA filler in the upper lip, injected using a threading technique.

**FIGURE 4:**
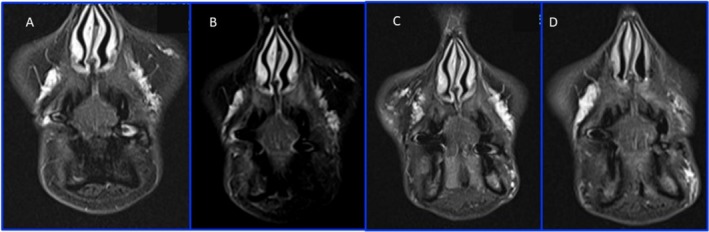
MRI: Coronal T2 TIRM in I (A), II (B), e III (C and D) MRI, showing the persistence of HA filler in the pyriform recess 24 months after the first treatment.

**FIGURE 5:**
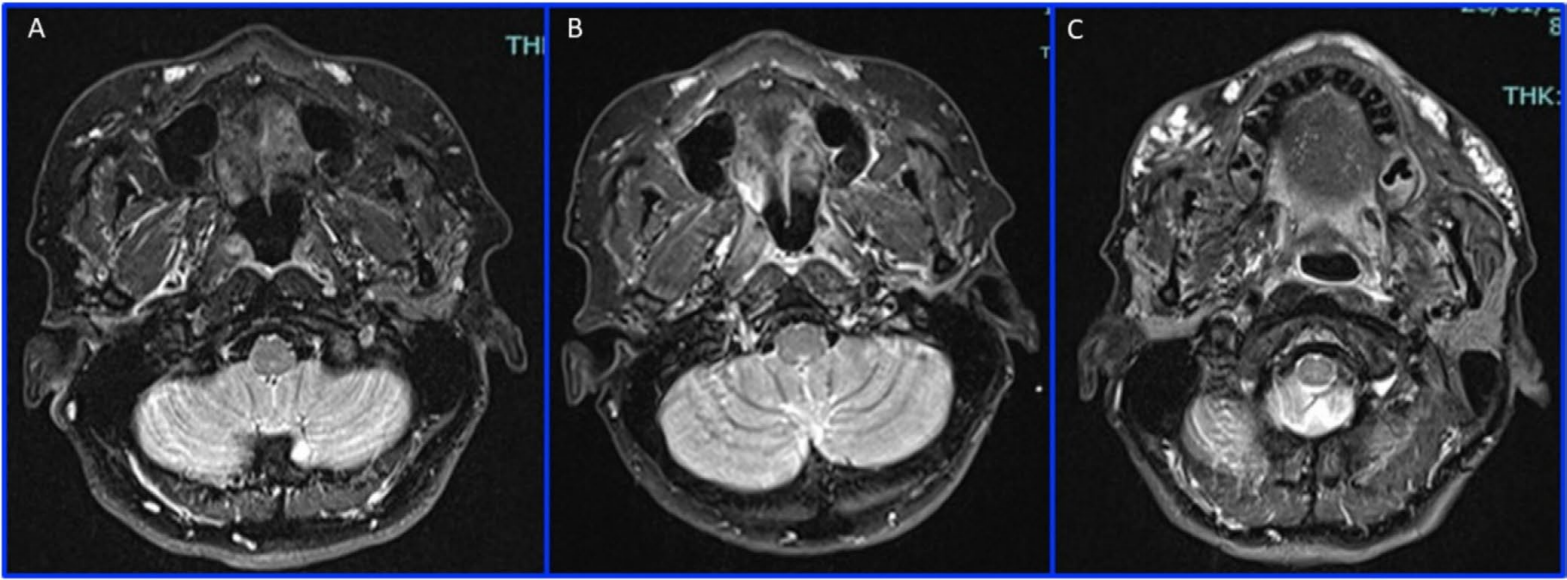
MRI: Axial T2 TIRM in I (A), II (B), e III (C) MRI, showing the persistence of HA filler in the pyriform recess 24 months after the first treatment.

**FIGURE 6:**
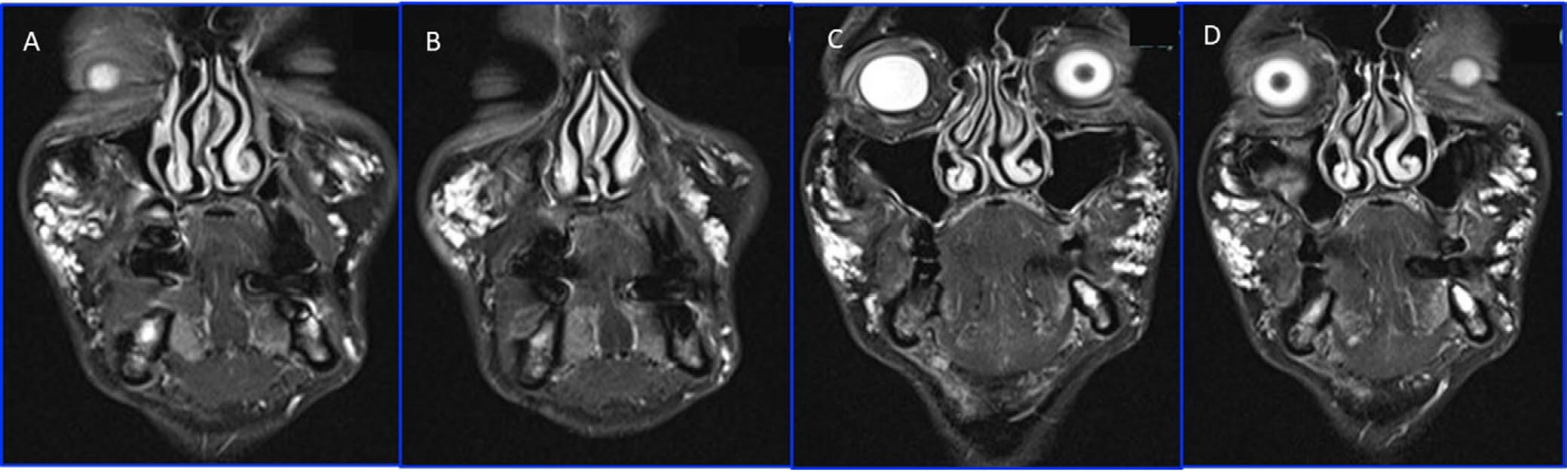
MRI: Coronal T2 TIRM of III MRI, showing HA filler in the zygomatic and cheek region.

The patient's MRI images revealed she underwent treatments with HA filler (Figures 1, 2, 3, 4, 5, 6). The signal of hyaluronic acid closely follows the water signal because of its composition and its hydrophilic nature [5]. Talking with the patient, we understood she lost a lot of facial volume because of aging and, above all, because of cancer treatments, in particular, following radiation therapy (RT) [6]. The first treatment was performed at the beginning of 2019 (product unknown) and the targets were the cheek, the nasolabial fold, and the upper lip; the second one was performed at the beginning of 2024 with Neofound (LOVE COSMEDICAL srls—Via Toniolo 9, 57,022 Castagneto Carducci, ITALY) containing Sodium Hyaluronate/Hyaluronic Acid HIGH molecular weight (1.500 < HA < 2.000 KDA) 24%, 107 Sodium Hyaluronate/Hyaluronic Acid LOW molecular weight (155 < HA < 230 KDA) 9%, Niacinamide, Glycine, Proline, BDDE, Lidocaine chlorhydrate 3%, using a 27G needle in the supra‐periosteal plane for mid‐face volume loss.

## 4 Outcome and Follow‐Up

The patient underwent MRI three times: the first 3 years after the first treatment with HA filler, the second 3 years and 6 months after the first treatment with HA filler, and the third 5 years after the first treatment with HA filler, that is a few days after the second facial treatment. Author 1, the radiologist, interpreted all the MRIs and made a direct comparison of the appearances of the deposits over the 24‐month period. The aim of our case report was:

– to focus on the appearance of HA filler in different locations, sites of bone resorption, and skeletal remodeling;
– to evaluate the longevity of the first filler implant and its MRI appearance over a 5‐year period after injection;
– to discover possible migrations of filler to other fat compartments.

We could not see a significant difference between the first and the second MRI (performed more than 6 months later), and in the third MRI, the filler was also well represented (Figure 6 and Table 1).

**TABLE 1:** MRI findings at various times after the first treatment with HA filler.

Time from the first treatment with HA filler	MRI findings
3 years	Persistence of HA filler implant located in the pyriform recess/nasolabial fold and upper lip
3 years and 6 months	Unchanged
5 years	Unchanged

## 5 Discussion

Magnetic resonance images demonstrated the persistence of HA filler in the zygomatic area and upper lip five years after injection without migration. Our work explored two different fields of interest. The first concerns the consequences of radiation therapy on the facial structures and the possible use of filler treatment to overcome these structural changes. Because of earlier diagnosis and newer effective therapies, cancer patients are showing an increase in overall survival; however, cancer survivors often face long‐term sequelae of anticancer treatment. Maintaining patients' quality of life is of paramount importance and it must be a target for clinicians; this can be achieved by a multidisciplinary treatment approach, including aesthetic treatments to improve patients' body image [7]. One of the most severe adverse effects of radiotherapy is osteoradionecrosis, an inflammatory condition resultant from the bone ionizing radiation. This radiation results in irreversible damages to the osteocytes and the microvascular system, with a progressive decrease of the microvascularization [6]. The tissue becomes hypovascular, hypocellular, and hypoxic. All these features prevent bone healing, and it can proceed to a necrosis with or without infection. These bone changes result from injury to the remodeling system (osteocytes, osteoblasts, and osteoclasts), causing atrophy [6]. Bone structures play an important role in inducing the typical “aged face” [8]: indeed, the bones of the facial mass (and therefore of the splanchnocranium) and those of the neurocranium (such as the frontal) constitute the craniofacial bone support. The facial filler can be used to counteract the effects that the actinic treatment causes on the bone support of the face. Just as the patient who underwent breast cancer surgery is offered plastic surgery to obtain breast harmonization, the patient who underwent facial radiation therapy should be offered a facial filler. Facial beauty and attractiveness are important intercultural social concepts and tend to dictate how individuals are judged and treated [9]. Moreover, injectable fillers are also used for the correction of disabling volumetric fat loss in HIV‐associated facial lipoatrophy, Parry Romberg syndrome, and post‐surgical and post‐traumatic facial disfiguring [10]. Our second field of interest was the evaluation of the filler's persistence in the subcutaneous adipose tissue. There are relatively few publications (about 30) regarding magnetic resonance imaging of facial dermal fillers. In one study, HA was reported to last for up to 12 years depending on the location and product in some patients [5]. Master wrote a case report to focus on the MRI appearance of HA injected over a 27‐month period [11]. The aim of our case report was to focus on the MRI appearance of HA injected over a 5‐year period. The signal of hyaluronic acid closely follows the water signal because of its composition and its hydrophilic nature [5]. Hyaluronic acid is a naturally occurring polysaccharide in healthy soft tissue, which binds the collagen and elastic fibers to provide intercellular stability. The injected HA combines with natural HA in the soft tissue, binds water due to its hygroscopic nature, and also induces new collagen formation [10]. Because of its high‐water content, HA filler appears strongly hyperintense on T2 W and TIRM sequences and hypointense on T1 W sequences. Turbo inversion recovery magnitude (TIRM) is an inversion recovery MRI pulse sequence; employing this sequence allows selective nulling (making certain materials appear dark) of specific tissue (e.g., inversion time can be set for fat suppression and to obtain a STIR sequence) [12]. This sequence is able to exalt the HA filler in the context of subcutaneous adipose tissue. The first and the second MRIs performed showed well‐defined linear threads of HA signal in the nasolabial fold and in the upper lip. The last MRI performed showed the persistence of the linear threads of HA signal above‐mentioned (in pyriform recesses and upper lip) and the presence of HA signal also in the zygomatic region and in the lateral cheek fat compartment region, bilaterally. Longevity seems to be related to the location, depth of filler placement, and volume of HA injected [13]. In Master study [11], HA injected in the superficial fat compartment of the lateral cheek should maintain HA signal for at least over 27 months. A factor contributing to this may be the total volume of HA injected in the lateral face, which was greater than that used for other areas of augmentation. Another factor may be the lack of mobility and subcutaneous placement in the lateral face, leading to the persistence of filler [14]. Our study demonstrates the persistence of HA filler in the zygomatic area and upper lip 5 years after injection. Decreased mobility of the face due to surgery and radiotherapy can be one of the reasons for the longevity of the filler in this patient. Most complications like erythema, bruising, and hypersensitivity do not require radiological evaluation. Occasionally, severe and acute complications like local soft tissue necrosis, blindness, and cerebral infarct may occur due to vascular occlusion. Acute blindness with glabella and nasolabial fold injection of hyaluronic acid is possible [15]. Long‐term complications are related to the injected filler itself and delayed host response. These complications include foreign body granulomas, abscesses, migration of filler, and tissue necrosis [10]. Filler migration is a potential complication following the injection of multiple fillers [16]. For example, injecting between the two laminae of the deep temporal fascia, the filler can migrate into the Bichat. With the increasing popularity of multiple filler injections, migrated granulomas should be an essential differential diagnosis for newly growing facial lumps. It is important for all physicians to be aware that complications by dermal fillers can occur in locations other than the planned injected sites [17]. Filler migration is a common indication for evaluation with MRI. Poor injection technique has been thought to cause filler migration. Although migration is not typical of any particular filler, permanent fillers (typically silicone) are more likely to migrate because of their long‐lasting permanence in the body. They may migrate through lymphatic or hematogenous routes and may mimic a malignant pathology of distant organs or granulomatous skin conditions. MRI is able to detect migrated facial fillers even in the absence of clinical suspicion and denial of a history of filler injection [10].

In this case, there was no evidence that HA had migrated to other fat compartments over our study period. Strong points of our case report are the third MRI was performed just a few days following the second treatment (looking forward to future investigations); the first and the third MRI were performed with the same device; the three MRIs were obtained with an analogous protocol of study. The first filler implant was found to be unchanged in appearance on sequential MRI. Ultrasound is an alternative imaging modality for dermal fillers; however, its shortcomings include its operator dependability and its poor reproducibility [10]; U.S. images are difficult to interpret by others at a later time; moreover, HA appearance changes over time, and it is more difficult to identify once integrated. MRI imaging provides a satisfying “snapshot” of the entire face [5]. Depending on availability, we can choose to use MRI or high‐frequency ultrasound to assess the location and volume of the injected facial fillers and to evaluate filler‐related complications. MRI has excellent soft tissue discrimination capability and the ability to provide anatomic, quantitative, and functional information. Traditionally, authors performing facial rejuvenation injections divided the facial fat into the superficial and deep fat layers separated by the superficial musculoaponeurotic system (SMAS). The SMAS is a fibrous network that connects the periosteum, the muscles of facial expression, the platysma, and the fascia of the parotid gland with the dermis [18]. Because of its high soft tissue resolution, MRI allows correct identification and localization of filler with respect to the muscle, parotid gland, and facial skeleton [10]. MRI has an excellent ability to detect soft tissue inflammation, abscess, and also foreign material in the soft tissues [19]. Our MRI examination protocol is aligned with the one employed by Mundada [10] and it is more complete than this. Often, injectable facial fillers are detected incidentally on cross‐sectional imaging studies. The incidentally detected facial filler poses a diagnostic challenge because the patient may forget or deny the history of filler injection (due to social taboo), or may not know what type of filler was used [20]. Moreover, a radiologist may be asked to evaluate the complications, extent, and location of a known facial filler injection. Therefore, radiologists need to be familiar with the imaging features of injectable fillers in order not to confound these with true pathology or vice versa in order not to miss true pathology obscured by filler injections. It is imperative for the radiologist to remain abreast of the injectable facial fillers, the injection procedures, and their complications to avoid misdiagnosis and unnecessary biopsy [10]. Foreign body granulomas, non‐granulomatous inflammation, and abscess associated with facial fillers may show increased uptake on FDG PET‐CT; this uptake is attributed to increased glycolysis in activated inflammatory cells [21]. The increased FDG uptake by facial fillers may pose a diagnostic challenge in head and neck cancer and melanoma patients by mimicking a new primary or recurrence, especially if the injection is performed between two follow‐up checks and the history is denied or simply forgotten [10].

## 6 Conclusion

Facial filler injection can be a valid tool to slacken, even in the long term, the effects of bone remodeling that occur in patients with oral cavity tumors and head and neck tumors who undergo radiotherapy treatment. MRI evidence of the longevity of HA injected into the nasolabial fold, zygomatic region, cheek region, and upper lip at 5 years from injection was well demonstrated in this case study. The longevity of HA implants into the lateral cheek fat and in the zygomatic region will be evaluated in future follow‐up MRI. Our MRI images showed no evidence of migration. Further MRI studies with larger cohorts, documenting the long‐term pattern of degradation of HA fillers, with differing properties, are needed. The present investigation also aimed to inform radiologists of the imaging features of injectable fillers in order not to confound these with true pathology or vice versa in order not to miss true pathology obscured by filler injections.

## Author Contributions

**Gloria Bettini:** conceptualization, data curation, writing – original draft. **Ferdinando De Negri:** data curation, formal analysis, visualization. **Roberto Amore:** formal analysis, funding acquisition, visualization. **Sergio Rexhep Tari:** conceptualization, data curation. **Antonio Scarano:** visualization, writing – original draft.

## Ethics Statement

The authors have nothing to report.

## Consent

Written informed consent was obtained from the patient to publish this report in accordance with the journal's patient consent policy.

## Conflicts of Interest

The authors declare no conflicts of interest.

## Data Availability

The data that support the findings of this study are available in the manuscript.
